# Middle cerebral artery blood velocity and end-tidal carbon dioxide responses to moderate intensity cycling in children, adolescents, and adults

**DOI:** 10.1152/japplphysiol.00688.2023

**Published:** 2024-09-12

**Authors:** Max E. Weston, Alan R. Barker, Owen W. Tomlinson, Jeff S. Coombes, Tom G. Bailey, Bert Bond

**Affiliations:** ^1^Faculty of Health and Life Sciences, Public Health and Sports Sciences, Children’s Health and Exercise Research Centre, University of Exeter, Exeter, United Kingdom; ^2^Physiology and Ultrasound Laboratory in Science and Exercise, School of Human Movement and Nutrition Sciences, The University of Queensland, Brisbane, Australia; ^3^School of Nursing Midwifery and Social Work, The University of Queensland, Brisbane, Australia

**Keywords:** age, cerebral blood flow, exercise, kinetics

## Abstract

This study investigated the middle cerebral artery blood velocity (MCAv) response to constant work-rate moderate-intensity cycling exercise in 21 children (9.3 ± 0.8 yr), 17 adolescents (12.3 ± 0.4 yr), and 20 young adults (23.6 ± 2.4 yr). Participants completed an incremental ramp test to exhaustion on a cycle ergometer to determine maximal oxygen uptake and gas exchange threshold (GET) before completing three 6-min transitions at a moderate intensity (90% GET) on separate visits. On each visit, bilateral MCAv was measured by transcranial Doppler ultrasonography and breath-by-breath end-tidal carbon dioxide ($\text{P}{\text{ET}}_{{\text{CO}}_{2}}$) via a metabolic cart. Data were ensemble-averaged for each participant and analyzed using a monoexponential model. Absolute MCAv was significantly higher throughout exercise in children and adolescents compared with adults (*P* < 0.001). Children had a significantly lower relative increase in MCAv from baseline (∼12%) compared with adolescents (∼20%) and adults (∼18%, *P* < 0.040). All adolescents and adults had a monoexponential rise in MCAv and $\text{P}{\text{ET}}_{{\text{CO}}_{2}}$, but this was observed in only eight children. Children and adolescents had a significantly faster MCAv time constant (τ, 12 ± 6 and 14 ± 8 s, respectively) compared with adults (27 ± 9 s, *P* < 0.001). MCAv τ was positively associated with faster $\text{P}{\text{ET}}_{{\text{CO}}_{2}}$ τ in adolescents (*r* = 0.70, *P* = 0.002) but not in children (*r* = −0.20, *P* = 0.640). Time- and amplitude-based response parameters of MCAv kinetics were significantly associated with $\text{P}{\text{ET}}_{{\text{CO}}_{2}}$ kinetics in adults (*r* = 0.50–0.74, *P* ≤ 0.025), but not in children (*r* = −0.19 to −0.48, *P* > 0.227). These findings suggest that the transition from childhood to adulthood impacts the MCAv response to exercise and the relationships between $\text{P}{\text{ET}}_{{\text{CO}}_{2}}$ and MCAv kinetics during exercise.

**NEW & NOTEWORTHY** This is the first study to find that children have smaller increases in Δ%MCAv (∼12%) during moderate-intensity exercise compared with adolescents and adults (∼18%–20%). Furthermore, MCAv kinetics were significantly faster in children and adolescents, compared with adults. MCAv kinetic responses were significantly and positively associated with $\text{P}{\text{ET}}_{{\text{CO}}_{2}}$ kinetics in adults, but not in children. These novel data also suggest that the regulatory role of $\text{P}{\text{ET}}_{{\text{CO}}_{2}}$ on MCAv during exercise begins to strengthen during adolescence.

## Graphical abstract

**Figure:**
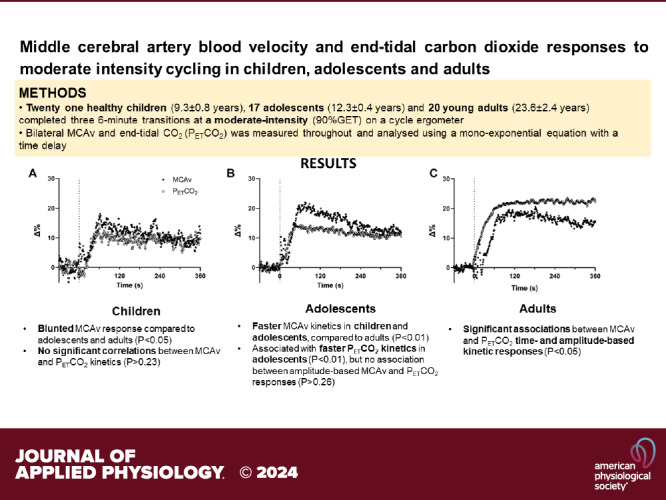

## INTRODUCTION

Exploring the kinetic response of middle cerebral artery blood velocity (MCAv) to constant work-rate exercise allows insight into time-based parameters, which are not possible from studying amplitude-based differences at fixed time points during exercise. In a seminal study by Billinger et al. (1), the MCAv kinetic response to moderate intensity exercise, performed at 45–55% of heart rate reserve on a recumbent stepper, was successfully modeled using a monoexponential model with a time delay. Subsequently, a smaller MCAv amplitude and a slower kinetic response (a greater time constant, τ) were observed in healthy older adults (aged ∼70 yr), compared with healthy young adults (aged ∼25 yr) (2), and a lower MCAv amplitude to moderate intensity exercise has been observed in stroke patients (1, 3). Collectively, these studies suggest that this measurement and analytical technique is sensitive to healthy aging and cerebrovascular disease. More recently, the same monoexponential approach has been used to characterize the MCAv response during moderate- and heavy-intensity upright cycling exercise using the exercise intensity domains paradigm (4, 5) in healthy young adults (6). While the MCAv kinetic response has been investigated in the context of aging during later life, the kinetic response of MCAv during exercise has yet to be investigated in children and adolescents. In addition to higher levels of resting cerebral blood flow (CBF) during childhood (7–9), children have been observed to have a blunted CBF response to exercise (10, 11). Indeed, during incremental exercise, MCAv increases by ∼10–15% (∼10–14 cm/s) from baseline in prepubertal children, which is almost half of that observed in both young adults and adolescents (∼20–30%, 18–23 cm/s) (10, 11). However, no study to date has directly compared the MCAv response to moderate-intensity exercise in children, adolescents, and adults.

In addition to an altered MCAv response to exercise in children compared with adults and adolescents, differences in the regulation of these responses have also been observed. While CBF is regulated through complex interactions between partial pressures of arterial blood gases [particularly carbon dioxide (${\text{Pa}}_{{\text{CO}}_{2}}$)], blood pressure, cerebral metabolism, sympathetic activity, and cardiac output (12, 13), a growing body of evidence suggests that changes in ${\text{Pa}}_{{\text{CO}}_{2}}$ are the primary regulator of CBF at rest (14) and during incremental exercise in adults (15). Although the intensity-dependent changes in MCAv during incremental exercise are positively associated with the intensity-dependent changes in end-tidal carbon dioxide ($\text{P}{\text{ET}}_{{\text{CO}}_{2}}$—used as a noninvasive surrogate of ${\text{Pa}}_{{\text{CO}}_{2}}$) in adults (10, 11), no significant associations have been observed between changes in MCAv and $\text{P}{\text{ET}}_{{\text{CO}}_{2}}$ during incremental exercise in prepubertal children (10, 11). These data suggest that the contribution of ${\text{Pa}}_{{\text{CO}}_{2}}$ in regulating MCAv during exercise is different between children and adults.

Our previous work has also observed that the relationships between exercise-induced changes in MCAv and $\text{P}{\text{ET}}_{{\text{CO}}_{2}}$ during ramp incremental exercise begin to strengthen during adolescence, compared with prepuberty (11), suggesting that the transition from childhood to adulthood influences the regulation of MCAv during exercise. However, a limitation of these data is that the use of incremental exercise does not allow a thorough investigation into the regulation of MCAv during exercise to define exercise intensity domains, as the work rate is constantly changing. Indeed, a key strength of analyzing the kinetic responses of physiological variables to constant work-rate exercise is that it allows insight into the underlying physiological control processes by investigating the temporal responses of other key variables to exercise, which would not be possible from studying steady-state responses alone (1). To date, no study has investigated the MCAv and $\text{P}{\text{ET}}_{{\text{CO}}_{2}}$ kinetic responses to moderate-intensity exercise in adults, adolescents, and children, an investigation that would provide detailed insight into the regulatory role of $\text{P}{\text{ET}}_{{\text{CO}}_{2}}$ during exercise.

Therefore, the aims of this study were to: *1*) compare the MCAv and $\text{P}{\text{ET}}_{{\text{CO}}_{2}}$ responses to moderate intensity cycling in children, adolescents, and adults and *2*) explore the relationships between MCAv and $\text{P}{\text{ET}}_{{\text{CO}}_{2}}$ kinetic responses to moderate-intensity exercise in children, adolescents, and adults. It was hypothesized that *1*) children would have smaller increases in MCAv during exercise compared with adolescents and adults and *2*) the relationships between MCAv and $\text{P}{\text{ET}}_{{\text{CO}}_{2}}$ kinetics would strengthen with increasing age group.

## METHODS

### Sample Size Calculation

This study was powered to detect a large effect size of the MCAv τ between children, adolescents, and adults. Although no study has investigated the MCAv kinetic response to exercise in children or adolescents, a large-very large effect size has been observed for the age-related differences in MCAv τ to moderate intensity exercise between young (23–25 yr) and older adults (65–67 yr) (*d* = 1.25–1.67) (2). Based upon a power of 0.8, alpha (α) of 0.05 and an anticipated effect size of 1.0 (between large and very large effect), a sample size of 17 participants per group was required. We aimed to recruit 20 participants in each group to account for participant drop-out and difficulty in obtaining an adequate MCAv signal in some participants.

### Participants

The data collection formed part of a wider study exploring MCAv responses to exercise in a range of populations, with some data published elsewhere (6, 11, 16), but the data presented here have not been previously published.

Twenty-one children (aged 8–10 yr, 10 males and 11 females), 17 adolescents (aged 12–14 yr, 10 males and 7 females), and 20 young adults (aged 19–28 yr, 10 males and 10 females) were recruited for this study using convenience sampling, with participant characteristics presented in Table 1. Child and adolescent participants were recruited from a local school in Devon, United Kingdom, and adult participants were recruited from the University of Exeter community. Following approval from the Sport and Health Sciences Ethics Committee, University of Exeter (190327/B/01), written informed consent was obtained for all adult participants. For the children and adolescents, written participant assent was obtained alongside written informed parental/guardian consent. Participants were initially screened for the study exclusion criteria, which included contraindications to maximal exercise, current use of any supplement or medication known to influence blood vessel function, and current or previous metabolic, cardiovascular, or cerebrovascular disease.

**Table 1.** Participant characteristics

	Children (*n* = 21)	Adolescents (*n* = 17)	Adults (*n* = 20)
Age, yr	9.3 ± 0.8^a,b^	12.3 ± 0.4^b,c^	23.5 ± 2.5^a,c^
Stature, cm	135.6 ± 6.6^a,b^	152.3 ± 9.4^b,c^	173.5 ± 9.3^a,c^
Body weight, kg	32.0 ± 8.8^a,b^	45.5 ± 9.4^b,c^	70.7 ± 12.5^a,c^
V̇o_2max_, L/min	0.96 ± 0.18^a,b^	1.63 ± 0.49^b,c^	2.71 ± 0.65^a,c^
V̇o_2max_, mL/kg^1.09^/min	22.6 ± 3.5^a^	25.9 ± 7.0	26.4 ± 5.7^a^
Peak power, W	78 ± 13^a,b^	141 ± 32^b,c^	286 ± 67^a,c^
GET, L/min	0.58 ± 0.10^a,b^	0.88 ± 0.28^b,c^	1.28 ± 0.31^a,c^
GET, % V̇o_2max_	60 ± 6^a,b^	54 ± 8^b,c^	48 ± 6^a,c^
Moderate-intensity power output, W	26 ± 5^a,b^	48 ± 15^b,c^	82 ± 24^a,c^
Moderate-intensity power output, %peak power output	34 ± 6^a^	34 ± 5^c^	29 ± 5^a,c^

All data shown as means ± SD. V̇o_2max_, maximal oxygen uptake; W, watts; GET, gas exchange threshold; ^a^*P* < 0.05 children vs. adults. ^b^*P* < 0.05 children vs. adolescents. ^c^*P* < 0.05 adolescents vs. adults.

### Experimental Protocol

Participants visited the laboratory on four separate occasions. On the first visit, participants completed a ramp incremental test to exhaustion on a cycle ergometer (Lode Paediatric Corival for children; Lode Excalibur for adolescents and adults, Lode, Groningen, the Netherlands). Participants then completed three experimental visits on separate days.

### Ramp Incremental Exercise

Following 3 min of seated rest on the cycle ergometer, participants completed a ramp incremental test to exhaustion at a ramp rate of 7–10 W/min (children), 10–20 W/min (adolescents), and 20–30 W/min (adults) to induce volitional exhaustion within ∼8–10 min (11). Participants were requested to maintain a consistent cadence between 70 and 90 revolutions per minute (rpm). Exhaustion was deemed to have been reached when the cadence fell below 70 rpm for five consecutive seconds, despite strong verbal encouragement from study investigators. Participants then rested for 10 min on the ergometer before completing a supramaximal verification test at 105% of their ramp test peak power until exhaustion (17, 18). Breath-by-breath pulmonary oxygen uptake (V̇o_2_), carbon dioxide production (V̇co_2_), and minute ventilation (V̇_E_) were collected using a leak-free facemask (Hans Rudolph) connected to a metabolic cart (Medgraphics Cardiorespiratory Diagnostics, UK), that was previously calibrated using a 3-L syringe and gases of known concentration. Data were exported as 10 s stationary averages and the V̇o_2max_ was defined as the highest 10 s averaged V̇o_2_ achieved during the ramp test or the verification bout (17). V̇o_2max_ data were also scaled allometrically using log-linear regression models to control for body size (19). This resulted in a scaling exponent (*b*) of 1.09, and V̇o_2max_ data were then scaled using a power function ratio (Y/X^b^). The V̇o_2_ corresponding to the gas exchange threshold (GET) was determined as the disproportionate increase in V̇co_2_ relative to V̇o_2_ (20) during the ramp test and verified by an increase in the ventilatory equivalent of oxygen (V̇_E_/V̇o_2_) without an increase in the ventilatory equivalent of carbon dioxide (V̇_E_/V̇co_2_). These were independently verified by more than one investigator.

### Experimental Visits

Participants completed three separate moderate-intensity bouts, each on separate days, at the same time of day (±1 h), with ≥24 h between visits. All visits were completed in a mean ± SD (range) of 18 ± 7 (6-29), 14 ± 7 (6–30), and 14 ± 6 (6–26) days in children, adolescents, and adults, respectively. Participants were asked to arrive at the laboratory following a ≥2 h fast and having avoided caffeine (37), alcohol (31), and vigorous exercise (21) for the 24 h preceding each visit, and verbally confirmed that they had adhered to the pretest instructions.

On each visit, participants completed 3 min of stationary, seated rest on the cycle ergometer, before an instantaneous transition to 6 min of constant work-rate moderate intensity cycling, completed at a consistent cadence between 70 and 90 rpm. The power output was designed to elicit a V̇o_2_ corresponding to 90% GET for each participant, which was determined from the linear relationship between work rate and V̇o_2_ during the ramp test, adjusted for a 30 s mean response time (38), and is presented in Table 1. Ninety percent GET was selected to reflect our previous work investigating MCAv kinetics in adults (6) and to preserve the work-rate in the children sample, where the work rate corresponding to GET can be low.

Due to data loss, one child and one adolescent participant only had complete data for two moderate-intensity bouts. To try and improve the signal-to-noise ratio of the acquired data, five children were invited to complete a fourth moderate-intensity bout.

### Experimental Measures

MCAv was measured bilaterally in all participants on every visit using transcranial Doppler (TCD) ultrasonography (DWL, Compumedics, Germany). Insonation of the left and right MCA was performed from an initial depth of 45–50 mm using two 2 MHz probes, secured in place with an adjustable headset (DiaMon, DWL, Germany). The position and depth of the probes were recorded for each participant and replicated between days. MCAv and heart rate (HR) data were collected at 200 Hz using an analogue-to-digital converter (Powerlab; model: 8/30, ADInstruments) interfaced with a laptop computer and stored for off-line analysis (LabChart 8, ADInstruments). Breath-by-breath $\text{P}{\text{ET}}_{{\text{CO}}_{2}}$, V̇o_2_, V̇co_2_, and V̇_E_ data were also collected throughout (Medgraphics Cardiorespiratory Diagnostics, UK).

### Data Analyses

Mean MCAv data were exported as 1 s averages and time aligned to exercise onset. Following our observation that there is bilateral parity in the MCAv response, the left and right MCAv data were averaged together, and in instances where one signal was lost, the remaining unilateral measurement was used (16). It was not possible to scan the right MCAv in one adult participant, so only unilateral left MCAv data were used for this participant. MCAv data from each moderate-intensity bout were then ensemble-averaged with the corresponding repeat transitions for each participant, creating a single MCAv trace for moderate-intensity exercise in each participant.

Breath-by-breath V̇o_2_, V̇co_2_, V̇_E,_ and $\text{P}{\text{ET}}_{{\text{CO}}_{2}}$ data were linearly interpolated to 1 s, time aligned to exercise onset and also ensemble-averaged with the corresponding repeat transitions, creating a single response for each participant. V̇_E_/ V̇co_2_ was also calculated as 1 s data for each participant.

Baseline MCAv and $\text{P}{\text{ET}}_{{\text{CO}}_{2}}$ were taken as the 60 s of seated rest preceding exercise onset. MCAv and $\text{P}{\text{ET}}_{{\text{CO}}_{2}}$ responses were expressed as a relative change from baseline (Δ%) and averaged into 10 s stationary averages every 30 s during exercise, both in absolute and relative terms.

### Kinetic Analyses

The kinetic responses of MCAv and $\text{P}{\text{ET}}_{{\text{CO}}_{2}}$ data were analyzed using methods previously described (6). Ensemble-averaged MCAv and $\text{P}{\text{ET}}_{{\text{CO}}_{2}}$ data were baseline corrected for the 60 s preceding exercise onset and analyzed using a monoexponential model with a time delay (*Eq. 1*) using GraphPad Prism (GraphPad Software, San Diego, CA).

(*1*) $$\text{y}\left(\text{t}\right)=\Delta {\text{y}}_{A}\left(1-{\text{e}}^{-(\text{t}-\text{TD}/\tau )}\right),$$

where y(t) is the MCAv or $\text{P}{\text{ET}}_{{\text{CO}}_{2}}$ at a given time (t), Δy_A_ is the amplitude change of MCAv (MCAv_A_) or $\text{P}{\text{ET}}_{{\text{CO}}_{2}}$ ($\text{P}{\text{ET}}_{\text{C}{\text{O}}_{2}\text{A}}$) from baseline to its asymptote, TD is the time delay, and τ is the time constant.

The model was fit from the start of the monoexponential rise, until a deviation from the initial exponential amplitude was observed. This was verified by more than one researcher, in line with our previous methods (6), and recorded for each participant. MCAv_end_ and $\text{P}{\text{ET}}_{\text{C}{\text{O}}_{2}\text{end}}$ data were taken as the last 10 s of exercise. ΔMCAv and Δ$\text{P}{\text{ET}}_{{\text{CO}}_{2}}$ were calculated as the difference between the amplitude of the exponential rise, and the end exercise value. To determine the appropriateness of each model fit, the residuals of each fit were inspected, and the standard error of the τ was extracted. The test-retest repeatability of these methods from previously published work from our laboratory during moderate intensity exercise yielded coefficients of variation of 7.1%, 35.9%, and 8.7% for MCAv baseline, τ, and amplitude, respectively (6).

### Statistical Analyses

All data are presented as means ± standard deviation (SD). Statistical analyses were performed using SPSS version 26 (IBM, Armonk, NY) and GraphPad Prism, with statistical significance set a priori at an α-level of 0.05.

To first investigate if there was an effect of sex on MCAv responses to exercise, a two-way independent measures ANOVA analyzed the effect of sex (male vs. female) × age (child vs. adolescent vs. adult) on MCAv kinetics. Furthermore, a three-way mixed model ANOVA investigated the effect of sex × age × time, to investigate if sex influenced the absolute and relative MCAv response during exercise.

MCAv, Δ%MCAv, $\text{P}{\text{ET}}_{{\text{CO}}_{2}}$_,_ and Δ%$\text{P}{\text{ET}}_{{\text{CO}}_{2}}$ every 30 s during exercise were analyzed using a two-way mixed model analysis of variance (ANOVA), with time as the within-subject factor, and age group as the between subject factor. Differences in participant characteristics, ramp test responses, baseline, and kinetic parameters between age groups were explored using a one-way ANOVA, with age group as the between-subject factor. Effect sizes have been calculated and reported to support the use of the *P* value. For the ANOVA main and interaction effects, these were displayed as partial eta squared ($\eta_{p}^{2}$) and interpreted as <0.06 = small, 0.06–0.14 = moderate, and ≥0.14 = large (22). Significant differences from ANOVA tests were located using pairwise comparisons and interpreted using the *P* value and standardized effect sizes (*d*). An effect size (*d*) was interpreted as small if <0.5, moderate if 0.5–0.8, and large if ≥0.8 (22).

Correlations between MCAv and $\text{P}{\text{ET}}_{{\text{CO}}_{2}}$ kinetic parameters in children, adolescents, and adults were explored using Pearson’s correlation.

## RESULTS

### No Sex Differences in the Changes in Middle Cerebral Artery Blood Velocities During Exercise

There was no significant main effect of sex on the absolute MCAv response to exercise (*P* = 0.293, $\eta_{p}^{2}$ = 0.02), nor was there a significant sex × time interaction (*P* = 0.911, $\eta_{p}^{2}$ = 0.01) or sex × age × time interaction (*P* = 0.505, $\eta_{p}^{2}$ = 0.04). For Δ%MCAv during exercise, there was no significant main effect of sex (*P* = 0.756, $\eta_{p}^{2}$ < 0.01), sex × time interaction (*P* = 0.895, $\eta_{p}^{2}$ < 0.01), or sex × age × time interaction (*P* = 0.742, $\eta_{p}^{2}$ = 0.03). Furthermore, there was no significant effect of sex on any kinetic parameter of the MCAv response to exercise (all *P* ≥ 0.070, $\eta_{p}^{2}$ < 0.08). Therefore, male and female data have been combined across all age groups for analysis.

**Figure 1.**
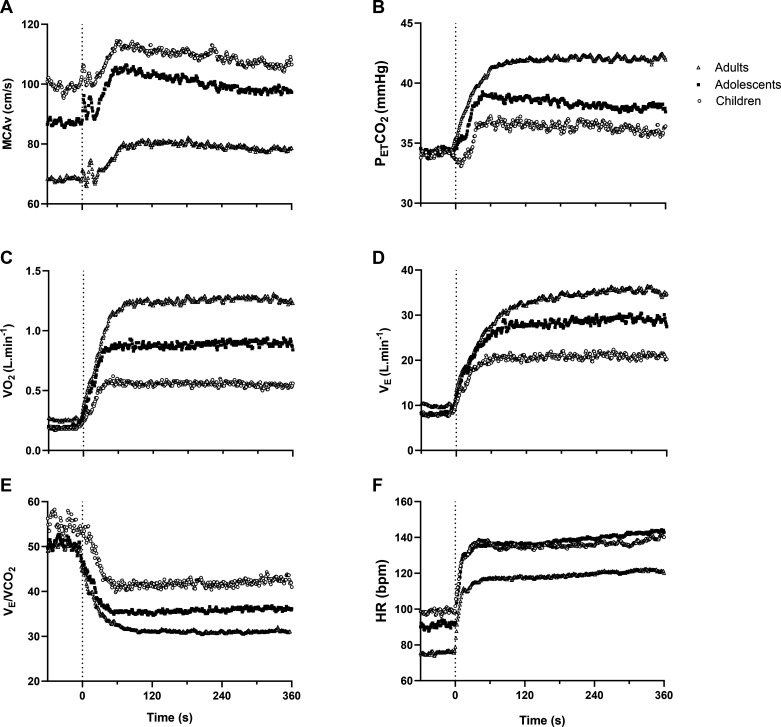
Group-averaged middle cerebral artery blood velocity (*A*), end-tidal CO_2_ (*B*), oxygen uptake (*C*), minute ventilation (*D*), V̇_E_/V̇co_2_ (*E*), and heart rate (*F*) responses to moderate intensity exercise in children (*n* = 21, open circles), adolescents (*n* = 17, closed squares), and adults (*n* = 20, open triangles). Dashed line indicates exercise onset.

Figure 1 shows the group-averaged MCAv, $\text{P}{\text{ET}}_{{\text{CO}}_{2}}$, V̇o_2,_ V̇_E_, HR, and V̇_E_/ V̇co_2_ responses for the whole sample, separated by age group.

### Differences in Middle Cerebral Artery Blood Velocities and End-Tidal Carbon Dioxide Responses in Adults, Adolescents, and Children

**Figure 2.**
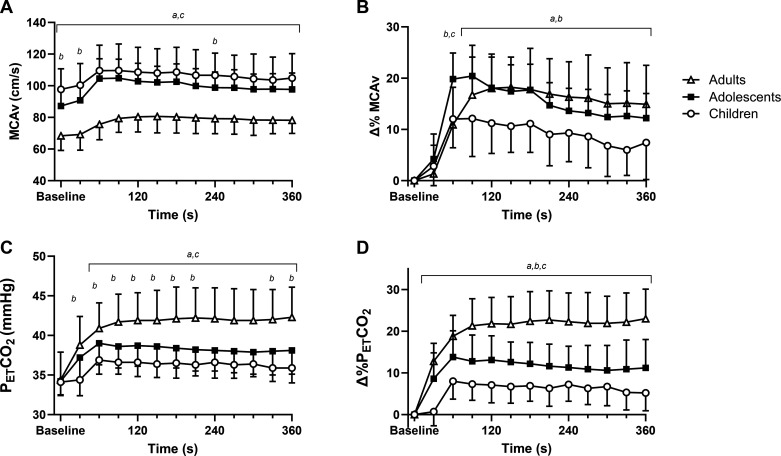
Middle cerebral artery blood velocity and end-tidal CO_2_ responses in absolute (*A* and *C*) and as a relative change from baseline (*B* and *D*) every 30 s during moderate intensity exercise in children (*n* = 21, open circles), adolescents (*n* = 17, closed squares), and adults (*n* = 20, open triangles). *^a^P* < 0.05 children vs. adults. *^b^P* < 0.05 children vs. adolescents. *^c^P* < 0.05 adolescents vs. adults. Data shown as means ± SD. Data analyzed using a two-way mixed model analysis of variance.

Figure 2 shows the absolute and relative change from baseline in MCAv and $\text{P}{\text{ET}}_{{\text{CO}}_{2}}$ every 30 s throughout moderate-intensity exercise.

### Middle Cerebral Artery Blood Velocity

Baseline MCAv was significantly higher in children (97.7 ± 13.0 cm/s) compared to adolescents (87.2 ± 10.0 cm/s, *P* = 0.005, *d* = 0.9) and adults (68.4 ± 9.3 cm/s, *P* < 0.001, *d* = 2.6), and in adolescents compared with adults (*P* < 0.001, *d* = 2.0). There was a significant age × time interaction for absolute MCAv during exercise (*P* < 0.001, $\eta_{p}^{2}$ = 0.17). Absolute MCAv was significantly higher throughout the exercise bout in children and adolescents compared with adults (*P* < 0.001). Absolute MCAv was also significantly higher in children compared with adolescents at 30 s (*P* = 0.013) and 240 s into the exercise bout (*P* = 0.043). For Δ%MCAv, there was a significant age × time interaction (*P* < 0.001, $\eta_{p}^{2}$ = 0.22). Δ%MCAv increased by 12.1 ± 7.4% in children, 20.4 ± 6.0% in adolescents, and 18.2 ± 5.9% in adults during moderate intensity exercise (Fig. 2*B*). From 90 s into the exercise bout until the end of exercise, children had a significantly lower Δ%MCAv compared to adolescents and adults (all *P* ≤ 0.040), and there were no differences between adolescents and adults. At 60 s, adolescents had a significantly higher Δ%MCAv compared to both children and adults (*P* < 0.001).

### End-Tidal Carbon Dioxide

Baseline $\text{P}{\text{ET}}_{{\text{CO}}_{2}}$ was not significantly different between any of the age groups (children: 34.1 ± 1.6 mmHg, adolescents: 34.2 ± 1.8 mmHg, adults: 34.4 ± 3.5 mmHg, *P* = 0.93, *d* ≤ 0.1). When comparing the absolute $\text{P}{\text{ET}}_{{\text{CO}}_{2}}$ response every 30 s during exercise, there was a significant age × time interaction (*P* < 0.001, $\eta_{p}^{2}$ = 0.40). From 60 s until the end of exercise, $\text{P}{\text{ET}}_{{\text{CO}}_{2}}$ was significantly greater in adults compared to both children and adolescents during moderate-intensity exercise (all *P* < 0.034). $\text{P}{\text{ET}}_{{\text{CO}}_{2}}$ was also significantly lower in children, compared with adolescents, from 30 s to 210 s during exercise, and in the last 60 s of moderate-intensity exercise (all *P* < 0.039, Fig. 2). Δ%$\text{P}{\text{ET}}_{{\text{CO}}_{2}}$ increased during exercise with a significant age × time interaction (*P* < 0.001, $\eta_{p}^{2}$ = 0.38). Throughout the 6-min exercise bout, Δ%$\text{P}{\text{ET}}_{{\text{CO}}_{2}}$ was significantly higher in adults compared with both children and adolescents (all *P* < 0.005) and significantly higher in adolescents compared with children (all *P* < 0.043).

### Kinetic Analyses

**Figure 3.**
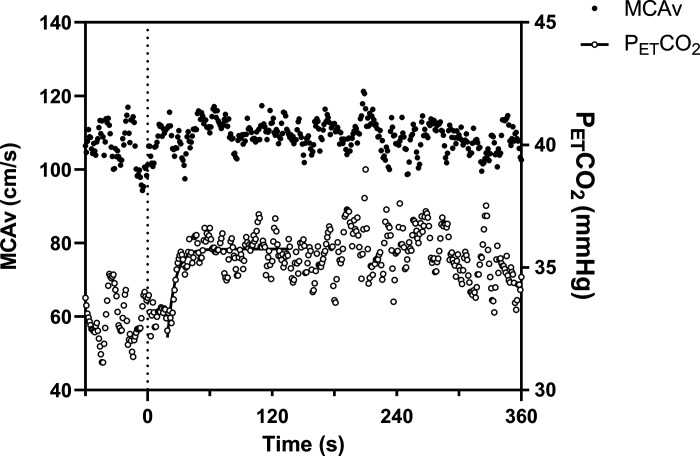
Middle cerebral artery (primary *y*-axis, closed circles) and end-tidal CO_2_ (secondary *y*-axis, open circles) response to moderate intensity cycling in one child participant. Data are shown as an ensemble average of three repeat transitions. Dashed line indicates exercise onset.

**Figure 4.**
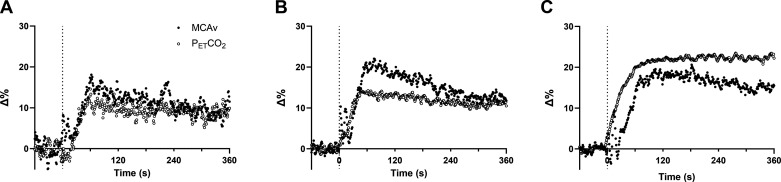
Relative change from baseline of middle cerebral artery blood velocity (MCAv, closed circles) and end-tidal CO_2_ ($\text{P}{\text{ET}}_{{\text{CO}}_{2}}$, open circles) in children (*n* = 8; *A*), adolescents (*n* = 17; *B*), and adults (C, *n* = 20). Dashed line indicates exercise onset.

All MCAv and $\text{P}{\text{ET}}_{{\text{CO}}_{2}}$ responses were able to be modeled using the monoexponential equation in adults and adolescents with appropriate model fits (τ standard error for MCAv: 3 ± 1 s and 2 ± 1 s, τ standard error for $\text{P}{\text{ET}}_{{\text{CO}}_{2}}$: 2 ± 1 s and 2 ± 2 s for adults and adolescents, respectively). However, in children, three did not have an exponential rise in MCAv and $\text{P}{\text{ET}}_{{\text{CO}}_{2}}$, eight had an exponential rise in MCAv but not $\text{P}{\text{ET}}_{{\text{CO}}_{2}}$, and two had an exponential rise in $\text{P}{\text{ET}}_{{\text{CO}}_{2}}$ but not MCAv (Fig. 3 shows an example). In those participants where an exponential rise was not observed, there was no detectable increase in MCAv and/or $\text{P}{\text{ET}}_{{\text{CO}}_{2}}$, and thus these responses could not be modeled (Fig. 3). Eight children (5 girls and 3 boys) showed an exponential increase in both MCAv (τ standard error for MCAv: 3 ± 1 s) and $\text{P}{\text{ET}}_{{\text{CO}}_{2}}$ (τ standard error for $\text{P}{\text{ET}}_{{\text{CO}}_{2}}$: 2 ± 1 s) at moderate-intensity exercise onset. Baseline MCAv was not significantly different in those who did (97.6 ± 7.8 cm/s) and did not (97.8 ± 15.6 cm/s, *P* = 0.981, *d* < 0.1) present exponential increases in MCAv and $\text{P}{\text{ET}}_{{\text{CO}}_{2}}$. There were also no differences in V̇o_2max_ between children who did (22.8 ± 2.4 mL/kg^1.09^/min) and did not (22.5 ± 4.1 mL/kg^1.09^/min, *P* = 0.877) present exponential rises in MCAv and $\text{P}{\text{ET}}_{{\text{CO}}_{2}}$. Therefore, kinetic analyses are presented for sample sizes of 20 adults, 17 adolescents, and 8 children. Figure 4 shows the relative change from baseline (Δ%) in MCAv and $\text{P}{\text{ET}}_{{\text{CO}}_{2}}$ in these children (Fig. 4*A*), adolescents (Fig. 4*B*), and adults (Fig. 4*C*).

### Middle Cerebral Artery Blood Velocity Kinetics

Table 2 shows the MCAv and $\text{P}{\text{ET}}_{{\text{CO}}_{2}}$ kinetic parameters during moderate-intensity exercise.

**Table 2.** MCAv and $\text{P}{\text{ET}}_{{\text{CO}}_{2}}$ kinetic responses to moderate intensity cycling in adults, adolescents and children

	Children (*n* = 8)	Adolescents (*n* = 17)	Adults (*n* = 20)	ANOVA	
				*P*	$\eta_{\text{P}}^{2}$
MCAv					
Baseline MCAv, cm/s	97.6 ± 7.8^a,b^	87.2 ± 10.0^b,c^	68.4 ± 9.3^a,c^	**<0.01**	0.62
MCAv τ, s	12 ± 6^a^	14 ± 8^c^	27 ± 9^a,c^	**<0.01**	0.40
MCAv TD, s	27 ± 7	24 ± 6	30 ± 11	0.09	0.11
MCAv_A_, cm/s	15.1 ± 7.7	17.8 ± 5^c^	13.1 ± 4.4^c^	**0.04**	0.14
MCAv_A_, Δ%	15.5 ± 7.7	20.5 ± 5.6	19.7 ± 7.6	0.23	0.07
MCAv_end_, cm/s	108.7 ± 11.6^a,b^	97.7 ± 10.3^b,c^	78.2 ± 8.3^a,c^	**<0.01**	0.63
MCAv_end_, Δ%	11.4 ± 7.7	12.2 ± 4.8	14.9 ± 7.6	0.33	0.05
Δ MCAv, %	4.1 ± 2.2^b^	8.3 ± 5.0^b,c^	4.8 ± 5.2^c^	**0.04**	0.14
Δ MCAv, onset s	152 ± 49^a^	165 ± 77^c^	242 ± 92^a,c^	**<0.01**	0.21
$\text{P}{\text{ET}}_{{\text{CO}}_{2}}$					
Baseline $\text{P}{\text{ET}}_{{\text{CO}}_{2}}$, mmHg	33.6 ± 1.8	34.2 ± 1.8	34.4 ± 3.5	0.77	0.01
$\text{P}{\text{ET}}_{{\text{CO}}_{2}}$ τ, s	11 ± 4^a^	13 ± 7^c^	30 ± 11^a,c^	**<0.01**	0.51
$\text{P}{\text{ET}}_{{\text{CO}}_{2}}$TD, s	22 ± 4^a^	17 ± 7^c^	5 ± 9^a,c^	**<0.01**	0.48
$\text{P}{\text{ET}}_{\text{C}{\text{O}}_{2}\text{A}}$, mmHg	3.7 ± 0.5^a^	4.8 ± 2.2^c^	7.8 ± 2.1^a,c^	**<0.01**	0.46
$\text{P}{\text{ET}}_{\text{C}{\text{O}}_{2}\text{A}}$, Δ%	11.0 ± 1.5^a^	14.1 ± 6.4^c^	23.1 ± 6.7^a,c^	**<0.01**	0.44
$\text{P}{\text{ET}}_{\text{C}{\text{O}}_{2}\text{end}}$, mmHg	36.6 ± 2.4^a^	38.1 ± 3.0^c^	42.3 ± 3.7^a,c^	**<0.01**	0.36
$\text{P}{\text{ET}}_{\text{C}{\text{O}}_{2}\text{end}}$, Δ%	9.0 ± 3.7^a^	11.2 ± 7.1^c^	23.1 ± 7.2^a,c^	**<0.01**	0.49
Δ $\text{P}{\text{ET}}_{{\text{CO}}_{2}}$, %	2.0 ± 3.3	2.8 ± 3.7^c^	0.0 ± 3.1^c^	**0.048**	0.14
Δ $\text{P}{\text{ET}}_{{\text{CO}}_{2}}$ onset, s	135 ± 37^a^	195 ± 85^c^	257 ± 91^a,c^	**<0.01**	0.25

MCAv, middle cerebral artery blood velocity; τ, time constant; TD, time delay; Δ%, relative change from baseline; MCAv_A_, amplitude of the exponential rise in MCAv; MCAv_end_, MCAv at the end of exercise; Δ MCAv, change in MCAv from the exponential amplitude to end of exercise; Δ MCAv onset, time point where MCAv deviated from the exponential rise; $\text{P}{\text{ET}}_{{\text{CO}}_{2}}$, end-tidal carbon dioxide; $\text{P}{\text{ET}}_{\text{C}{\text{O}}_{2}\text{A}}$, amplitude of the exponential rise in $\text{P}{\text{ET}}_{{\text{CO}}_{2}}$; $\text{P}{\text{ET}}_{\text{C}{\text{O}}_{2}\text{end}}$, $\text{P}{\text{ET}}_{{\text{CO}}_{2}}$ at the end of exercise; Δ $\text{P}{\text{ET}}_{{\text{CO}}_{2}}$, change in $\text{P}{\text{ET}}_{{\text{CO}}_{2}}$ from the exponential rise to end of exercise; Δ $\text{P}{\text{ET}}_{{\text{CO}}_{2}}$ onset, time point where $\text{P}{\text{ET}}_{{\text{CO}}_{2}}$ deviated from the exponential rise. Bold indicates significant ANOVA effect (*P* < 0.05). ^a^*P* < 0.05 children vs. adults. ^b^*P* < 0.05 children vs. adolescents. ^c^*P* < 0.05 adolescents vs. adults.

Baseline MCAv and MCAv_end_ were significantly higher in children compared with adolescents and adults, and in adolescents compared to adults (all *P* < 0.013, *d* ≥ 1.0). The absolute amplitude of the exponential rise in MCAv was significantly greater in adolescents compared to adults (*P* = 0.010, *d* = 1.0), with no difference between children and adolescents or adults (*P* ≥ 0.255, *d* < 0.5). When expressed as a relative change from baseline, there was no significant difference in Δ%MCAv between age groups (*P* = 0.234, *d* < 0.8), nor Δ%MCAv at the end of exercise (*P* = 0.330, *d* < 0.5). The exponential rise in MCAv was significantly quicker (smaller τ) in children and adolescents, compared to adults (*P* < 0.001, *d* > 1.5), with no difference between children and adolescents (*P* = 0.584, *d* = 0.3). MCAv TD was not significantly different between age groups (*P* = 0.085, *d* < 0.7). After an initial exponential rise in MCAv, MCAv fell from a steady state towards the end of the exercise bout. The magnitude of this fall (ΔMCAv) was significantly greater in adolescents compared to both children and adults (*P* ≤ 0.044, *d* ≥ 0.7), but was not different between children and adults (*P* = 0.749, *d* = 0.2). The onset of ΔMCAv occurred significantly earlier in children and adolescents, compared with adults (*P* ≤ 0.011, *d* ≥ 0.9), with no difference between children and adolescents (*P* = 0.705, *d* = 0.2).

### End-Tidal Carbon Dioxide Kinetics

$\text{P}{\text{ET}}_{{\text{CO}}_{2}}$ was not significantly different at baseline between age groups (*P* = 0.767, *d* < 0.3). The amplitude of the exponential rise in $\text{P}{\text{ET}}_{{\text{CO}}_{2}}$ and $\text{P}{\text{ET}}_{{\text{CO}}_{2}}$ at the end of exercise, both in absolute and relative terms, were significantly greater in adults, compared to children and adolescents (all *P* < 0.001, *d* ≥ 1.4). The τ of the exponential rise in $\text{P}{\text{ET}}_{{\text{CO}}_{2}}$ was significantly greater (slower) in adults, compared to children and adolescents (*P* < 0.001, *d* ≥ 1.8), and the TD was significantly smaller in adults compared to children and adolescents (*P* < 0.001, *d* ≥ 1.5). Similarly to MCAv, $\text{P}{\text{ET}}_{{\text{CO}}_{2}}$ did not maintain a steady state and fell during the exercise bout in some participants. The onset of this fall (Δ$\text{P}{\text{ET}}_{{\text{CO}}_{2}}$) occurred significantly earlier in children and adolescents, compared to adults (*P* < 0.028, *d* ≥ 0.7), and fell by a significantly greater magnitude in adolescents, compared to adults (*P* = 0.017, *d* = 0.8).

No differences in any $\text{P}{\text{ET}}_{{\text{CO}}_{2}}$ kinetic parameters were observed between children and adolescents (all *P* ≥ 0.095, *d* ≤ 0.8).

### Correlations Between Middle Cerebral Artery Blood Velocity and End-Tidal Carbon Dioxide Kinetics

**Figure 5.**
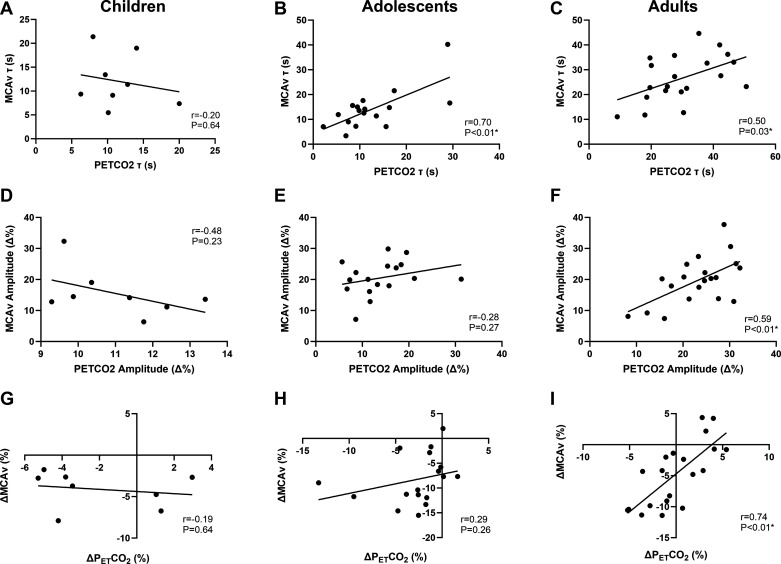
Relationships between MCAv and $\text{P}{\text{ET}}_{{\text{CO}}_{2}}$ time constant (children, *n* = 8, *A*; adolescents, *n* = 17, *B*; adults, *n* = 20, *C*), amplitude (children, *D*; adolescents, *E*; adults, *F*) and Δ (change from the initial exponential amplitude to end exercise, children, *G*; adolescents, *H*; adults, *I*). Data are analyzed using Pearson correlation.

Figure 5 shows the relationships between MCAv and $\text{P}{\text{ET}}_{{\text{CO}}_{2}}$ τ, amplitude (Δ%), and change (Δ%) in children, adolescents, and adults.

MCAv and $\text{P}{\text{ET}}_{{\text{CO}}_{2}}$ τ were significantly and positively correlated in adults and adolescents (*r* = 0.50, *P* = 0.025 and *r* = 0.70, *P* = 0.002, respectively), but were not significantly associated in children (*r* = -0.20, *P* = 0.640). The amplitude of MCAv and $\text{P}{\text{ET}}_{{\text{CO}}_{2}}$ were significantly and positively correlated in adults (*r* = 0.59, *P* = 0.006) but not in adolescents (*r* = 0.28, *P* = 0.273) or children (*r* = −0.48, *P* = 0.227). Similarly, ΔMCAv and Δ$\text{P}{\text{ET}}_{{\text{CO}}_{2}}$ were significantly and positively correlated in adults (*r* = 0.74, *P* < 0.001), but not in adolescents (*r* = 0.29, *P* = 0.260) or children (*r* = −0.19, *P* = 0.644).

Given the significant associations between Δ$\text{P}{\text{ET}}_{{\text{CO}}_{2}}$ and ΔMCAv in adults following the exponential rise, the adult sample was then split into those who did (*n* = 11), and those who did not (*n* = 9), present a fall in $\text{P}{\text{ET}}_{{\text{CO}}_{2}}$ during exercise. The ventilatory responses (V̇o_2_, V̇_E,_ and V̇_E_/V̇co_2_) to moderate-intensity exercise in these two groups are shown in Supplemental Fig. S1.

## DISCUSSION

The main findings of this study are that children have a blunted MCAv response to moderate-intensity exercise (∼12% increase in MCAv) compared to adolescents (∼20%) and adults (∼18%). This study is the first to investigate the kinetic response of MCAv during exercise in youth, and found significantly faster MCAv kinetics in children and adolescents, compared to adults. Furthermore, the kinetic response of MCAv to moderate-intensity exercise was not associated with $\text{P}{\text{ET}}_{{\text{CO}}_{2}}$ kinetics in children, but was significantly and positively associated in adults. In adolescents, the faster MCAv response at exercise onset was significantly correlated with faster $\text{P}{\text{ET}}_{{\text{CO}}_{2}}$ kinetics, but there were no significant associations between amplitude-based changes in $\text{P}{\text{ET}}_{{\text{CO}}_{2}}$ and MCAv.

### Middle Cerebral Artery Blood Velocity and End-Tidal Carbon Dioxide Responses to Moderate-Intensity Exercise

These data support previous findings during incremental exercise from our laboratory (11), and others (10), in that children have a smaller Δ%MCAv increase during exercise and a greater baseline MCAv compared with both adolescents and adults. This has led to suggestions that children have a reduced cerebrovascular reserve as a consequence of their elevated resting levels of CBF, compared to adolescents and adults (23, 27). However, there was no significant difference between baseline MCAv in children that did, and did not, present an exponential increase in MCAv and $\text{P}{\text{ET}}_{{\text{CO}}_{2}}$ in the present study. These data extend previous findings and suggest that the blunted MCAv response to exercise in children, compared with adolescents and adults, is attributed to more than simply an elevated baseline MCAv, potentially challenging the idea of a reduced cerebrovascular reserve in children. Furthermore, in order to properly investigate this hypothesis, a “maximal” CBF stimulus is needed to elucidate whether there is a reduced maximal vasodilatory capacity in children. Moderate-intensity exercise does not elicit maximal increases in MCAv (6). It is therefore possible that MCAv is being down-regulated in children during moderate-intensity exercise or that other factors are contributing to the reduced MCAv amplitude, rather than simply a reduced cerebrovascular reserve. Furthermore, the idea that prepubertal children have a reduced cerebrovascular reserve has been contested by data at rest, where Tallon et al. (39) observed that cerebrovascular reactivity to CO_2_ breathing was not different in prepubertal children and adults, and Talbot et al. observed that cerebrovascular reactivity (CVR) was not different in pre- compared with post-PHV youth (40).

In children and adolescents, there was a smaller $\text{P}{\text{ET}}_{{\text{CO}}_{2}}$ response to exercise, similar to observations during incremental exercise (10, 11, 36). There are a number of possible reasons underpinning this, including smaller CO_2_ storage and production in children (36), and greater ventilatory sensitivity to CO_2_ production during exercise (24). Despite this, adolescents experienced a similar increase in Δ%MCAv as adults during exercise. It has been suggested from magnetic resonance imaging data at rest that CVR to CO_2_ increases during adolescence (27). However, CVR to steady-state CO_2_ breathing was recently found to be similar in pre- and post-PHV youth (40), and developmental changes in CVR to CO_2_ remain unclear and are likely influenced by both the stimulus and imaging modality. Specifically, the study by Leung et al. (27) used BOLD MRI and 45 s blocks of hypercapnia, compared to 4 min of fixed concentration CO_2_ breathing, where there is more time for steady-state MCAv values to be attained. This is a particularly important consideration as the kinetic responses of MCAv to different stimuli, such as exercise (1, 2, 6) and CO_2_ breathing (39, 40), continue to gain more research interest, particularly in the context of aging- and maturation-related differences. Furthermore, it is likely that the MCAv response to exercise is multifaceted, given the dynamic changes observed in a number of key regulatory factors of CBF (15) and potentially altered relationships with changes in ${\text{Pa}}_{{\text{CO}}_{2}}$ and CBF between age groups (10, 39).

### Middle Cerebral Artery Blood Velocity and End-Tidal Carbon Dioxide Kinetic Responses to Moderate-Intensity Exercise

The present study observed a faster MCAv kinetic response (smaller τ) in children and adolescents, compared to adults, with no difference between children and adolescents. In their study, Ward et al. (2) observed slower MCAv kinetics and a reduced MCAv amplitude in older adults (∼70 yr) compared to younger adults (∼25 yr). Taken collectively, these data suggest that MCAv kinetics slow with increasing age from adolescence, into adulthood, and then further into older adulthood. However, all available data are cross-sectional in nature, and highlight the need for future longitudinal studies to investigate developmental and aging-related changes in MCAv kinetic responses to exercise.

One key observation from the present study was that only eight (out of 23) children presented an exponential rise in MCAv and $\text{P}{\text{ET}}_{{\text{CO}}_{2}}$, which could be modeled, with a number of children showing no discernible increase in MCAv at exercise onset (see Fig. 3 for an example). There were no differences in baseline MCAv and cardiorespiratory fitness between children who did and did not present an exponential rise in MCAv and $\text{P}{\text{ET}}_{{\text{CO}}_{2}}$, suggesting that these factors are not contributing to these altered response profiles observed in children. By contrast, all adolescent and adult responses were able to be modeled. While the MCAv kinetic response to exercise has never previously been modeled in children, Tallon et al. (39) characterized the MCAv kinetic response to CO_2_ breathing and were unable to model the response in six (out of 20) prepubertal children. In their study, children presented a significantly greater τ (∼42 s slower) to CO_2_ breathing compared to adults. This differs from the present findings during exercise, where MCAv τ in children was more than twice as fast as observed in adults. These data suggest that the dynamic adjustment of MCAv in children, and how it compares to adult responses, is highly influenced by the challenge presented to the cerebrovasculature, with CO_2_ breathing and exercise providing two very different stimuli. It is likely that this is underpinned by dynamic changes in a number of key factors that regulate CBF at exercise onset, including arterial blood gases, blood pressure, cardiac output, cerebral metabolism, and sympathetic nerve activity (15, 34, 35).

In addition, Tallon et al. (41) observed an uncoupling of the $\text{P}{\text{ET}}_{{\text{CO}}_{2}}$ and MCAv kinetic responses to CO_2_ breathing in prepubertal children, whilst these were closely aligned in adults. In agreement, we observed no significant association between MCAv and $\text{P}{\text{ET}}_{{\text{CO}}_{2}}$ kinetic responses, in terms of both time (τ) and amplitude (exponential rise and subsequent fall in MCAv) responses in children. Furthermore, this is evident in the example provided in Fig. 3, where MCAv does not change despite an exponential rise in $\text{P}{\text{ET}}_{{\text{CO}}_{2}}$, further highlighting the uncoupling of MCAv and $\text{P}{\text{ET}}_{{\text{CO}}_{2}}$ responses during exercise, even in those children for whom exponential modeling was not possible. These data agree with previous observations from our laboratory (11) and others (10) during incremental exercise, where the intensity-dependent changes in MCAv were not significantly associated with the intensity-dependent changes in $\text{P}{\text{ET}}_{{\text{CO}}_{2}}$. These novel data add to a growing body of evidence that suggests that ${\text{Pa}}_{{\text{CO}}_{2}}$ plays a limited role in the regulation of CBF during exercise in children. Ellis et al. (10) also observed no significant associations between changes in mean arterial pressure (MAP) and MCAv during incremental exercise in prepubertal children. As a result, the underpinning mechanisms for the increase in MCAv during exercise in some children and the lack of increase in others remain poorly understood. It is known that children have elevated cerebral oxygen consumption at rest, compared to adults (42), and the blunted MCAv response to exercise may reflect a reduced cerebral metabolic demand during exercise in children. Furthermore, the proportion of cardiac output delivered to the brain is 10% greater in prepubertal children (aged 8–10 yr) compared with adults (43). Tallon et al. (39) suggested that such changes in cardiac output may have an important role in CBF responses during hypercapnia in children, as the temporal responses of MCAv, $\text{P}{\text{ET}}_{{\text{CO}}_{2}}$, and MAP were poorly aligned during CO_2_ breathing. It is therefore possible that age-related differences in cerebral oxygen consumption and/or cardiac output responses to exercise contribute to the age-related differences in MCAv regulation observed in the present study, but this requires investigation.

By contrast, MCAv and $\text{P}{\text{ET}}_{{\text{CO}}_{2}}$ kinetic responses were significantly and positively associated in adults. These data support those from incremental exercise (10, 11), and the use of kinetic modeling to constant work-rate exercise is a strength of this study, allowing a unique insight into the underlying control mechanisms of MCAv during exercise. A large body of evidence suggests that changes in ${\text{Pa}}_{{\text{CO}}_{2}}$ are the primary regulator of the intensity-dependent changes in CBF during incremental exercise in adults (15), but the regulation during submaximal exercise is less clear. Smith et al. (44) observed similar increases in MCAv of ∼20% during sub-maximal recumbent exercise up to 60% W_max_ with (isocapnic) and without (poikilocapnic) a $\text{P}{\text{ET}}_{{\text{CO}}_{2}}$ clamp in healthy adults. These data suggest that the increase in MCAv during exercise occurs independently of increases in $\text{P}{\text{ET}}_{{\text{CO}}_{2}}$, contrary to the majority of observations during incremental exercise (15). The present data build on these findings, and suggest that, during upright cycling, both the time- and amplitude-based responses of MCAv are related to $\text{P}{\text{ET}}_{{\text{CO}}_{2}}$ kinetics in healthy adults. However, limitations in estimating ${\text{Pa}}_{{\text{CO}}_{2}}$ via $\text{P}{\text{ET}}_{{\text{CO}}_{2}}$ during exercise, detailed below, may confound comparisons between studies (25, 28, 36). Furthermore, exercise modality may also influence the relationships between $\text{P}{\text{ET}}_{{\text{CO}}_{2}}$ and MCAv during exercise, with data suggesting greater MCAv/$\text{P}{\text{ET}}_{{\text{CO}}_{2}}$ reactivity during upright compared to recumbent cycling (29, 44). Our findings may offer further support for the effect of exercise posture and modality, and suggest that ${\text{Pa}}_{{\text{CO}}_{2}}$ has a regulatory role in the MCAv response to upright constant work-rate cycling exercise in adults.

In our earlier work, we observed that MCAv did not always maintain a steady state during constant work-rate moderate-intensity exercise in healthy adults (6). Here, we extend these previous findings and can attribute the fall in MCAv during exercise to reductions in $\text{P}{\text{ET}}_{{\text{CO}}_{2}}$ during exercise. The fall in $\text{P}{\text{ET}}_{{\text{CO}}_{2}}$ in 11 adults (up to a 6% fall) was an unexpected observation during moderate-intensity exercise, but we show that this is not due to hyperventilation, given the similar and steady-state responses of V̇o_2_, V̇_E,_ and V̇_E_/V̇co_2_ in those who do and do not experience a fall in $\text{P}{\text{ET}}_{{\text{CO}}_{2}}$ (Supplemental Fig. S1). This was also observed in 14 adolescent participants, and the mechanisms responsible for this observation are unclear, but it could also be a consequence of alterations in the ${\text{Pa}}_{{\text{CO}}_{2}}$-$\text{P}{\text{ET}}_{{\text{CO}}_{2}}$ gradient during exercise (25, 28, 36). To explain these mechanisms, previous studies have shown that hyperthermia causes a reduction in both $\text{P}{\text{ET}}_{{\text{CO}}_{2}}$ and MCAv during prolonged exercise in adults, but this was also mediated via hyperventilation (32). The present study was performed in a temperature-controlled room, and the exercise was of moderate intensity for just 6 min, so exercise-induced changes in body temperature are unlikely to be underpinning this observation. It is possible that this is a consequence of beginning exercise from a stationary start (thus needing to overcome initial inertia), which was chosen for the present study given the low power outputs for moderate-intensity exercise in children, in an attempt to preserve any amplitude that may exist in this population. This is in contrast to using a “freewheel” period or slowly adjusting to the target work rate over a 30-s period, as performed in previous studies investigating MCAv kinetics that did not report a fall in MCAv (1, 2). This remains speculation and requires further investigation in protocol comparison studies. Previous work using an extended exercise duration (20 min) also observed a progressive fall in both MCAv and MAP during semirecumbent moderate-intensity cycling exercise in healthy young adults (45). Given the absence of MAP measurements in the present study, the influence of changes in MAP during exercise on the observed fall in MCAv cannot be discounted (45).

This was the first study to investigate the MCAv kinetic response to exercise in adolescents, who represent a group transitioning into adulthood. This study found significantly faster MCAv kinetics in adolescents, compared to adults, which were significantly associated with faster $\text{P}{\text{ET}}_{{\text{CO}}_{2}}$ kinetics at exercise onset. However, the amplitude of the exponential rise in MCAv was not significantly associated with the amplitude of the exponential rise in $\text{P}{\text{ET}}_{{\text{CO}}_{2}}$. Furthermore, following this initial exponential rise, the fall in MCAv was significantly greater in adolescents compared with both adults and children and was not associated with a fall in $\text{P}{\text{ET}}_{{\text{CO}}_{2}}$. The mechanisms underpinning both the increase and decrease in MCAv during moderate intensity in adolescents remain unexplained, but the present study shows these are unrelated to $\text{P}{\text{ET}}_{{\text{CO}}_{2}}$. Some evidence suggests that healthy adolescents have altered cerebral autoregulation compared to adults and a delayed return of CBF following acute changes in blood pressure (46). It is possible that a poorer ability to “buffer” the exercise-induced increases in blood pressure at exercise onset, especially given the stationary start and need to overcome the initial inertia, led to a greater initial increase, or “overshoot” in MCAv at exercise onset in adolescents, that decreases as the bout progresses. However, developmental changes in cerebral autoregulation remain poorly understood, and the study by Vavilala et al. (46) used transient hypotension at rest. There is a need for future research to investigate the relationships between changes in MAP, cardiac output, and MCAv during exercise in adolescents, as well as perform exercise of greater duration, to determine if MCAv attains a steady state or continues to fall. Previous work in healthy young adults observed that MCAv progressively fell by 13% during 20 min of semirecumbent cycling at 45–60% of heart rate reserve and only stabilized during the last 5 min of exercise (45). Furthermore, the fall in MCAv occurred alongside a progressive fall in MAP during exercise, but these data are not available in children or adolescents, highlighting the need for future investigations. Nevertheless, the present data support our previous data from incremental exercise (11), and suggest that the regulatory role of ${\text{Pa}}_{{\text{CO}}_{2}}$ begins to develop and strengthen during adolescence. This is supported by significant associations between MCAv and $\text{P}{\text{ET}}_{{\text{CO}}_{2}}$ τ in adolescents, contrary to children, but not amplitude-based responses, contrary to adults.

### Study Considerations

This is the first study to investigate the MCAv kinetic response during moderate-intensity exercise in children and adolescents and has a number of methodological strengths. These include the use of repeat transitions to enhance the signal-to-noise ratio of acquired data (1, 6), reflected by the strong confidence of the τ estimates. This is also the first study to explore the kinetic responses of MCAv and $\text{P}{\text{ET}}_{{\text{CO}}_{2}}$ simultaneously, allowing a greater insight into the regulatory role of $\text{P}{\text{ET}}_{{\text{CO}}_{2}}$ on exercise MCAv responses and how these are influenced by age. Finally, the use of the exercise intensity domains to prescribe work rate at the same relative intensity anchored around each individual’s GET (4) is an additional strength of the study. However, when expressed relative to V̇o_2max_, GET was significantly higher in children compared with adolescents and adults, and significantly greater in adolescents compared to adults. This meant that the moderate intensity power output, when expressed relative to maximum, was significantly higher in children and adolescents, compared with adults.

The present study found no influence of sex on any of the MCAv outcomes to moderate intensity exercise, which agrees with some previous research investigating MCAv responses to exercise in healthy children, adolescents and/or adults (6, 10, 11, 47), but is in contradiction to others during moderate and high-intensity interval exercise (2, 48). It is important to note that this study is likely underpowered to detect sex differences, with low sample sizes for some subgroups (3–11 participants). Sex hormones have been suggested to have an important effect on CBF, in particular during puberty (9, 23, 26); future research on larger sample sizes is required to further understand the roles and interactions of sex, maturation, and age on CBF responses to exercise.

The present study used TCD to measure cerebral blood velocity in the MCA. A strength of this approach is the ability to continuously and noninvasively measure cerebral blood velocity during whole-body movements, which has been widely used during exercise in children, adolescents, and adults (10, 11, 34). Furthermore, the excellent temporal resolution from TCD allows the application of kinetic modeling of the time and amplitude-based responses (1, 2, 6). However, TCD does not measure vessel diameter and is only an appropriate surrogate of CBF if vessel diameter remains constant (49). MCA is known to change diameter during marked alterations in $\text{P}{\text{ET}}_{{\text{CO}}_{2}}$ in adults (+15 mmHg, −13 mmHg) (49). During moderate-intensity exercise in the present study, the amplitude of increase of $\text{P}{\text{ET}}_{{\text{CO}}_{2}}$ was smaller than this across all age groups, so it is unlikely that changes in MCA diameter are underpinning the primary observations of this study. However, changes in MCA diameter may occur during steady state increases in $\text{P}{\text{ET}}_{{\text{CO}}_{2}}$ of as little as 4.5 mmHg in adults (50) and since vessel diameter was not measured in the present study, this cannot be excluded, particularly in pediatric populations, where limited MRI data is available.

The present study used $\text{P}{\text{ET}}_{{\text{CO}}_{2}}$ as a surrogate of ${\text{Pa}}_{{\text{CO}}_{2}}$, which forms an important limitation of the study. Although recent data shows that $\text{P}{\text{ET}}_{{\text{CO}}_{2}}$ provides an accurate estimation of ${\text{Pa}}_{{\text{CO}}_{2}}$ across a wide range of ${\text{Pa}}_{{\text{CO}}_{2}}$ levels at rest in healthy young adults (30), the relationships between $\text{P}{\text{ET}}_{{\text{CO}}_{2}}$ and ${\text{Pa}}_{{\text{CO}}_{2}}$ are weaker during constant work rate and incremental exercise, with $\text{P}{\text{ET}}_{{\text{CO}}_{2}}$ overestimating ${\text{Pa}}_{{\text{CO}}_{2}}$ during exercise (25, 28, 36). In particular, the $\text{P}{\text{ET}}_{{\text{CO}}_{2}}$-${\text{Pa}}_{{\text{CO}}_{2}}$ difference is influenced by breathing pattern and is increased with greater V̇co_2_ and V̇t and lower breathing frequencies during exercise (25). Given that ventilation also impacts CBF and MCAv responses to exercise (11, 33), limitations in estimating ${\text{Pa}}_{{\text{CO}}_{2}}$ from $\text{P}{\text{ET}}_{{\text{CO}}_{2}}$ may be confounding the correlations and interpretations presented in this study. Furthermore, in both children and adults, Ohuchi et al. (36) found a widening of the $\text{P}{\text{ET}}_{{\text{CO}}_{2}}$-${\text{Pa}}_{{\text{CO}}_{2}}$ difference during incremental exercise at the GET, but suggested that $\text{P}{\text{ET}}_{{\text{CO}}_{2}}$ was a better estimate of ${\text{Pa}}_{{\text{CO}}_{2}}$ during exercise in children. Nevertheless, $\text{P}{\text{ET}}_{{\text{CO}}_{2}}$ provides a commonly used and noninvasive estimate of ${\text{Pa}}_{{\text{CO}}_{2}}$ during exercise. Due to the challenges in securing accurate beat-to-beat fingertip MAP values during whole body exercise, particularly in youth, a further limitation of the present study is the absence of blood pressure measurements during exercise, as alterations in MAP are an important CBF regulator (15). Further research exploring the relationships between MCAv, MAP, and cardiac output responses to exercise will further elucidate the physiological mechanisms underpinning the MCAv responses to exercise, particularly in children and adolescents, where $\text{P}{\text{ET}}_{{\text{CO}}_{2}}$ seems to have a diminished regulatory role compared with adults.

## CONCLUSIONS

This is the first study to explore the MCAv response to moderate-intensity exercise in children, adolescents, and adults, and found that children have a smaller increase in Δ% MCAv during exercise compared to adolescents and adults. This study found that MCAv kinetics were significantly faster in children and adolescents compared with adults. In adults, MCAv kinetic responses were significantly and positively associated with $\text{P}{\text{ET}}_{{\text{CO}}_{2}}$ kinetics, but were not significantly associated in children. In adolescents, the faster MCAv kinetics were associated with faster $\text{P}{\text{ET}}_{{\text{CO}}_{2}}$ kinetics, but the amplitude-based changes in MCAv during exercise were not associated with $\text{P}{\text{ET}}_{{\text{CO}}_{2}}$ changes. These novel data suggest that children may have distinctly different mechanisms of MCAv regulation during exercise. These data also suggest that the regulatory role of ${\text{Pa}}_{{\text{CO}}_{2}}$ on MCAv begins to develop during the transition from childhood to adulthood.

## DATA AVAILABILITY

Data will be made available upon reasonable request.

## SUPPLEMENTAL MATERIAL

10.6084/m9.figshare.26031478 Supplemental Fig. S1: https://doi.org/10.6084/m9.figshare.26031478.

## GRANTS

This work was supported by the QUEX Institute (University of Queensland and University of Exeter).

## DISCLOSURES

No conflicts of interest, financial or otherwise, are declared by the authors.

## AUTHOR CONTRIBUTIONS

M.E.W., A.R.B., J.S.C., T.G.B., and B.B. conceived and designed research; M.E.W. performed experiments; M.E.W., A.R.B., O.W.T., and B.B. analyzed data; M.E.W., A.R.B., O.W.T., and B.B. interpreted results of experiments; M.E.W. prepared figures; M.E.W. drafted manuscript; A.R.B., O.W.T., J.S.C., T.G.B., and B.B. edited and revised manuscript; M.E.W., A.R.B., O.W.T., J.S.C., T.G.B., and B.B. approved final version of manuscript.
